# Patients’ Preferences Regarding Traditional Chinese Medicine for the Treatment of Chronic Obstructive Pulmonary Disease: Protocol for a Mixed Methods Study

**DOI:** 10.2196/75426

**Published:** 2025-12-02

**Authors:** Shaonan Liu, Tongtong Wu, Yan Yu, Yingkai Liu, Jing Wang, Xiaoli Chen, Xinfeng Guo

**Affiliations:** 1 The Second Affiliated Hospital of Guangzhou University of Chinese Medicine, Guangdong Provincial Hospital of Chinese Medicine Guangzhou China; 2 The Second Clinical College of Guangzhou University of Chinese Medicine Guangzhou China

**Keywords:** traditional Chinese medicine, preference, mixed methods, discrete choice experiment, chronic obstructive pulmonary disease

## Abstract

**Background:**

Chronic obstructive pulmonary disease (COPD) is now one of the top 3 causes of death worldwide. Traditional Chinese medicine (eg, herbal prescriptions and acupuncture), which has a long history of managing respiratory diseases, has shown positive effects in COPD management by alleviating dyspnea, improving lung function, and reducing the risk of acute exacerbations. Patients’ values and preferences are undeniably important in medical decision-making and may affect treatment outcomes and patient adherence.

**Objective:**

This study aims to investigate the preferences for traditional Chinese medicine of patients with COPD, the clinical outcomes they are concerned about, and the trade-offs involved in evaluating the factors that influence treatment selection, such as clinical effectiveness, cost, and adverse effects.

**Methods:**

We will first update previous evidence through a systematic review of randomized controlled trials, examining the efficacy, safety, cost-effectiveness, and patient satisfaction of traditional Chinese medicines for COPD. Subsequently, an exploratory sequential mixed methods study will be conducted comprising qualitative and quantitative components. In this design, qualitative findings are collected first to inform and guide the subsequent quantitative data collection. Semistructured interviews will be conducted to explore in depth the preferences of patients with COPD for traditional Chinese medicine. Insights from these interviews will then be used to design questionnaires that quantitatively investigate the relative importance of different factors influencing patients’ treatment decisions. Data integration will take place by connecting and interpreting the results from both the qualitative and quantitative steps, providing a comprehensive understanding of patient preferences.

**Results:**

This study was approved by the ethics committee of Guangdong Provincial Hospital of Traditional Chinese Medicine on November 6, 2023 (ZM2023-405). A total of 18,188 articles published after 2016 were initially identified in English- and Chinese-language databases. The outline of the semistructured interview guide for this study has been developed. Further clinical evidence updates, qualitative interviews, and discrete choice experiments are still ongoing and will be completed by April 2026.

**Conclusions:**

This mixed methods study might provide important insights into the preferences of patients with COPD for traditional Chinese medicine, assessing trade-offs among efficacy, safety, cost, and other key factors that influence treatment decisions. This study is expected to deepen the understanding of patient-centered decision-making in the treatment of COPD. The findings are anticipated to guide clinical practice, inform policy development, and optimize the integration of traditional Chinese medicine with respiratory care.

**International Registered Report Identifier (IRRID):**

DERR1-10.2196/75426

## Introduction

“Patients’ values and preferences,” as a broad term, is defined as including patient perspectives, beliefs, expectations, and goals for their health and life, including the process that patients go through in weighing the potential benefits, harms, costs, and burdens associated with different treatment or disease management options [1]. Patients’ preferences are based on subjective perceptions and values and are influenced by previous experiences, which may affect treatment outcomes and patient adherence; thus, their importance in medical decision-making is unquestionable [2,3].

Many years ago, patients’ preferences were suggested to be included in clinical practice guidelines [4]. Patient and public representatives are recommended to engage in the guideline development process. On the basis of the requirements of evidence-based medicine (EBM) and narrative medicine, adequate consideration of patients’ needs or understanding of specific treatments is a strong guarantee of scientific and humanized clinical practice guideline development [5]. In addition, understanding patients’ preferences is an important expression of respect for the basic principles of medical ethics [6]. However, patient representatives’ participation [7,8] is insufficient to reflect individual patients’ preferences. Individuals often vary widely in their preferences, even when recommendations are based on rigorous evidence, due to differences in values, experiences, and cultural backgrounds, among other things. Shared decision-making (SDM) is when clinicians and patients participate collaboratively in health decisions after fully discussing treatment options, benefits, and harms, considering the patients’ values, preferences, and circumstances [9]. Applying SDM in clinical practice may result an individualized therapeutic regimen for each patient. Achieving active decision-making requires work by the health care provider and patient. The health care provider teams need to be proactive, and the patient needs to be prepared. Patients increasingly want to be active in clinical decision-making, especially younger patients and those with higher educational levels [10,11]. They want to be informed by their physicians and prefer to learn about their treatment options early and discuss the benefits and drawbacks. Knowing this information can help patients weigh the treatment effects, side effects, and costs so that treatment decisions can be matched to their preferences. Therefore, it remains important for health care providers to recognize patients’ preferences.

Chronic obstructive pulmonary disease (COPD) is a heterogeneous lung condition characterized by chronic respiratory symptoms due to airway or alveoli abnormalities that cause persistent, often progressive, airflow obstruction. COPD is now one of the top 3 causes of death worldwide, and 90% of these deaths occur in low- and middle-income countries [12,13]. It has resulted in a significant economic and social burden that is projected to increase in the coming decades, with substantial morbidity and mortality worldwide [14]. Medication adherence is a major challenge in chronic disease management. A surprising fact is that medication adherence is lower in patients with COPD than in those with other diseases [15], with an average medication adherence of only 60% reported in several studies [16]. Studies have shown that patients with COPD with higher medication adherence have fewer hospitalizations and spend less on treatment compared to those with lower medication adherence [17]. Most of the drugs available for COPD are delivered via inhalers [18,19]. The use of an inhaler requires several steps and differs by device. Patients should receive additional training and education on the use of inhalers, which significantly increases the burden of their disease management [20,21]. Studies indicate that “convenience-related” attributes, such as ease of use, appropriate shape, and low chance of misuse, are among the factors that influence patient preference for inhalers. Furthermore, according to recent work by the World Health Organization and other organizations, inhaled medications are scarcely available and mostly unaffordable for patients with COPD in low- and middle-income countries [22].

Traditional Chinese medicine (TCM) has been used to manage respiratory diseases for thousands of years and has been comprehensively and systematically evaluated for its clinical effectiveness in treating COPD. Evidence shows that TCM therapies, including acupuncture and tai chi, can help improve clinical outcomes such as lung function, quality of life, breathlessness, and exercise capacity in patients with COPD and are safe and reliable [23-25]. Chinese herbal medicines have shown potential benefits for stable or acute exacerbations of COPD [26-28].

The results of a pilot study conducted by our team suggest that the preference of patients with COPD for TCM may be influenced by factors such as clinical efficacy, side effects, and cost [29]. The findings need to be validated in larger samples. In addition, patient perceptions with regard to specific interventions have not been investigated. In this study, we focus on exploring the preferences of patients with COPD during clinical decision-making. *Preference* is defined as patients’ perceptions of TCM for the treatment of COPD, their appraisal of factors for treatment scenarios, and the balance of harms and benefits within or between interventions.

The discrete choice experiment (DCE) is a stated-preference method originating from Lancaster’s economic theory of value and McFadden’s random utility theory, and it has since been widely applied in health care research [30,31]. DCEs can integrate measures of multiple attributes to obtain results closer to real choices, which can adequately predict actual consumer behavior, being generally used in health care preference surveys today [32,33]. There are currently no studies that have used DCE to investigate the preferences of patients with COPD for TCM. Therefore, we will use mixed methods, including both qualitative and quantitative (DCE) methods to obtain more comprehensive information. We hypothesize that clinical practice will be improved by incorporating patients’ preferences. Our objective is to investigate patients’ preferences for TCM for the treatment of COPD; the clinical outcomes they are concerned about; and the trade-offs for the factors of treatment scenario options, such as clinical effectiveness, cost, and side effects. The main research questions are as follows:

1. What is patients’ perception (such as effect, side effects, and treatment duration) of TCM for COPD?
2. Which clinical outcomes are of most concern for patients, and which factors are important for treatment scenario options?
3. Which factors will influence patients’ options in terms of the available evidence for specific therapies (eg, efficacy, safety, or economic factors)?

## Methods

### Study Design

**Figure 1:**
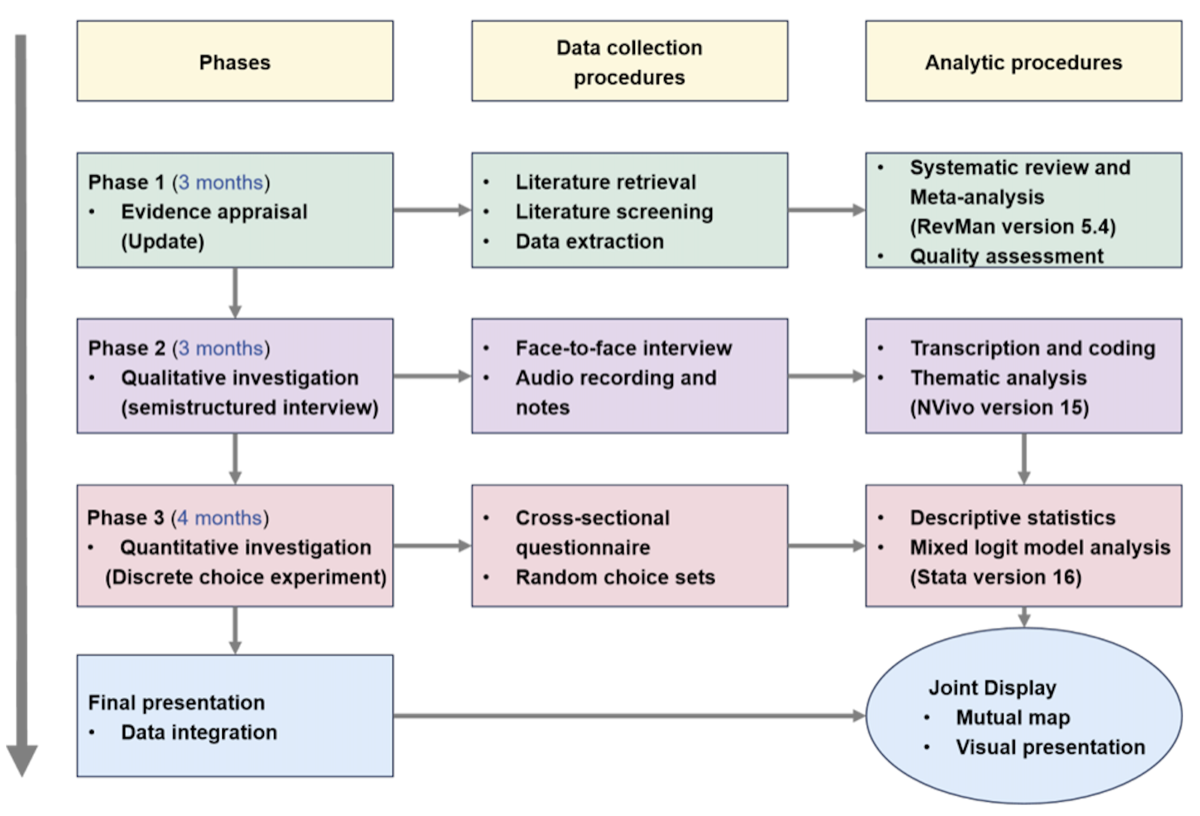
Procedural diagram of the exploratory sequential mixed methods study on patients’ preferences for traditional Chinese medicine in the treatment of chronic obstructive pulmonary disease.

An exploratory, sequential mixed methods study will be conducted involving a qualitative and quantitative study (DCE) [34]. In an exploratory sequential design, data are first collected and analyzed through qualitative investigation, followed by further exploration of themes derived from the qualitative data via a quantitative investigation. The results from both sets of data are then integrated and interpreted [35]. The attributes of the DCE will be identified from a literature review and the qualitative survey based on previous pilot study results [29]. The results of the qualitative and quantitative study phases will be compared to determine the degree of consistency. This study, which is expected to be completed within 1 year, will be divided into three phases, which are illustrated in the procedural diagram (Figure 1): (1) an update of the existing evidence [23] on the efficacy, safety, economic benefits, and patient satisfaction related to TCM in treating COPD (phase 1; 3 months); (2) a qualitative investigation involving semistructured interviews to explore the perception of COPD, expectations of interventions, and attitudes toward TCM, among other aspects (phase 2; 3 months); and (3) a cross-sectional DCE, which aims to understand participants’ preferences for the TCM interventions and assess the relative importance of different attributes of treatment scenarios through a questionnaire (phase 3; 4 months).

### Participants

Patients aged ≥18 years with a diagnosis of COPD based on The Global Initiative for Chronic Obstructive Lung Disease criteria assessed by a clinical specialist [19] who currently prescribes or has previously prescribed TCM and is able to communicate in Mandarin will be included. Patients with evidence of mental illness, who are judged unable to cooperate or complete the survey, or who are receiving TCM to treat other diseases but not COPD will be excluded. There are no restrictions on the stage and severity of the condition, and a broad range of participants is expected to be enrolled to draw comprehensive conclusions.

Preferences can be diverse in patients from different hospital backgrounds. Recruitment of participants will be conducted based on sufficiently broad background characteristics. Therefore, patients will be recruited from various hospitals. Additional participants will be recruited through newsletters, flyers, or advertisements or when they return for a hospital visit. Participants who have taken part in the interviews will also be invited to take part in the subsequent survey.

All participants will be informed about the study and will sign a consent form before undergoing study procedures.

### Setting

This study will be conducted in a private room at the hospital rather than in the clinic so that patients can relax and openly discuss the interview questions or complete the questionnaires.

### Sample Size

The sample size for the semistructured interviews will be determined through data saturation, and representative participants will be identified using a purposeful sampling strategy. Data saturation will be reached when no additional novel themes emerge from interviews with participants after data analysis and a sufficient number of participants with different characteristics have been interviewed. Between 10 and 20 potential participants will be included in each site considering the diversity of hospitals.

Multiple factors should be considered in the sample size estimation of the DCE survey, such as the heterogeneity of participants, the structure of the DCE questions, the items of the questionnaire, and the expected precision of the results. A sufficiently large sample size is crucial for capturing significant preference changes in the observed population and distinguishing different preferences among subgroups. However, large-scale studies require substantial resources. The thumb formula is a method proposed by Johnson and Orme [36] to calculate the minimum sample size. Considering the limited resources of this study, we used this formula to calculate the minimum sample size required for the DCE main-effects model. In this formula, 500 is a fixed variable, *c* is the maximum number of levels in any attribute, *t* is the number of DCE questions per questionnaire, and *a* refers to the number of options included in each DCE question:

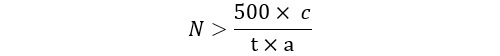



### Data Collection

#### Phase 1

A systematic review will be conducted following the PRISMA (Preferred Reporting Items for Systematic Reviews and Meta-Analyses) guidelines. We will search 3 English-language databases (PubMed, Embase, and Cochrane Library) and 4 Chinese-language databases (Chinese Science and Technology Journal Database, Chinese Biomedical Literature Database, Wanfang, and China National Knowledge Infrastructure) using the following key search terms: “Chronic Obstructive Pulmonary Disease,” “Chinese Medicine,” and “Randomized controlled trials” (more details on the search strategy can be found in Multimedia Appendix 1). Randomized controlled trials exploring the efficacy, safety, and economic benefits of TCM for the treatment of COPD will be included. The initial screening will be carried out independently by 2 researchers who will read the titles, abstracts, and full texts separately and in strict accordance with the inclusion and exclusion criteria. The inclusion and exclusion criteria will be based on a previous systematic review [23]. Data extraction forms will be predesigned using EpiData (version 3.1; EpiData Association), and 2 reviewers will independently extract data from the included studies, checking the data after extraction and resolving disagreements on the extracted data through discussion or third-party assistance.

#### Phase 2

Two researchers will conduct the interviews and collect the data. Both have received training on interviewing techniques and data collection procedures and have experience communicating with patients. To ensure objectivity and minimize potential bias, patient recruitment and scheduling will be handled by a separate research coordinator. The interviewers will not be involved in these preliminary stages and will remain blinded to participant identities until the interviews begin. Before the initiation of the interviews, patients will be briefed on the background and main content of the study and promised that the management of the interview audio recordings will protect their privacy to gain their trust. They will also sign an informed consent form. The open-ended interview questions will be designed by the research team based on a comprehensive review of the literature and findings from our preliminary research (Table 1). The content of the interview outline will be flexibly adjusted according to each individual interview, and the participants will be encouraged to actively express their thoughts in a positive way, choices, and expectations regarding the health care process to obtain richer information from the interviews. Participants can also add any new ideas that are important to them but not covered in the current framework. Basic characteristics (gender, age, educational level, and condition duration) will be collected via a questionnaire. The full interview will be audio recorded, and notes will be taken. The interviews will be conducted in a quiet and private venue to ensure that the interview process is not disturbed and will take 60 minutes on average.

**Table 1:** Topic guide for the semistructured interview used to investigate treatment preferences for traditional Chinese medicine among patients with chronic obstructive pulmonary disease (COPD).

Topic	Relevant questions
Perception of the disease	What are your concerns about COPD and what do you want to know about it? What are your expectations for COPD in the future?
Treatment history	When and under what circumstances did you start receiving traditional Chinese medicine treatment during the course of your illness?
Perception of the treatment	What is your perceived effectiveness of traditional Chinese medicine? Comparing to other treatments you had received throughout the course of illness, what is your perception (such as effect, side effects, and treatment duration) of traditional Chinese medicine for COPD? What clinical outcomes are you more concerned about?
Factors affecting treatment options	Which factors are important for treatment scenario options?

Interviewees will be assigned numbers to protect their privacy, and the audio recordings will be transcribed verbatim by the research team within 24 hours after the interview. Transcripts will be individually coded by the interviewers, and potential themes will be elicited accordingly and then entered into a database (NVivo Plus; version 12; Lumivero). The interviewers will be involved in the coding process and check the coding. Major differences in coding will be discussed and resolved by experienced qualitative researchers.

#### Phase 3

This study’s questionnaire is divided into 3 parts. The first part collects basic personal information of the participants, including age, gender, educational level, primary caregiver, per capita monthly family income, and type of health insurance. The second part focuses on disease and treatment information, such as disease type, stage, and duration and treatment plan. The third part includes preference questions based on identified attributes and levels. Using SAS (version 9.4; SAS Institute), choice sets will be generated via efficient design (*D*–efficiency). When there are too many choice sets, they will be evenly distributed across several questionnaire versions to reduce patients’ burden. Each choice set offers 2 alternatives (options A and B) plus an exit option. To ensure survey quality, the consistency of patients’ pre- and postchoices will be checked by repeating a choice set question and swapping options A and B. Inconsistent choices will be deemed as invalid data.

A pilot survey will be conducted with a small target group to test the comprehensibility of the questionnaire and the rationality of the DCE design. On the basis of this feedback, several discussions will be held to modify and adjust the language and questions of the survey. The same group of participants will then be selected for a second test to gradually improve the reliability and validity of the questionnaire. On the basis of standardized research steps, the research team will strictly control the quality of the questionnaire. The pilot surveys will be completed by the researchers through face-to-face on-site communication to reduce errors and improve the quality of the questionnaire.

Formal research will be carried out in several TCM and Western medicine hospitals. Researchers will distribute and collect paper questionnaires face-to-face to ensure data quality. Before the survey, they will explain the precautions in detail. While patients are filling out the questionnaire, the researchers will answer any questions that arise on the spot.

### Data Analysis

#### Phase 1

A systematic review will be conducted using Review Manager (version 5.4; The Cochrane Collaboration). Risk of bias will be assessed according to the revised Cochrane risk-of-bias tool for randomized trials [37]. The Grading of Recommendations Assessment, Development, and Evaluation system will be used to estimate the certainty of the evidence.

#### Phase 2

Data analysis and data collection will take place simultaneously. Theoretical saturation will be determined to terminate the interviews when the information from the last interview has been analyzed and no new themes and ideas have been found. The transcribed texts will be read repeatedly for familiarization and line-by-line coding. Initial codes will generate potential themes. The interview transcripts will be analyzed using thematic analysis, which is an inductive method for identifying, analyzing, and reporting patterns within the data [38]. The NVivo qualitative analysis software will be used to facilitate and manage the analysis. After analyzing and processing the interview transcripts, we will provide a subset of participants with the research findings or interpretations to verify whether they accurately reflect the intended meanings of the participants.

#### Phase 3

The analysis will commence using descriptive statistics, summarizing sociodemographic and clinical characteristics as means and SDs for continuous variables and as frequencies and proportions for categorical variables. The core analysis of the DCE data will used a mixed logit model. This model assesses how the different attributes and levels influence patient choices, with the choice outcome as the dependent variable and the attribute levels as independent variables. The model estimates a regression coefficient (β) for each attribute, where a significant β denotes a meaningful impact on preference, with its sign and magnitude indicating the direction and relative strength of influence, respectively. The associated SD of β quantifies the heterogeneity in preferences for that attribute among participants. All analyses will be conducted using Stata (version 16.0; StataCorp).

#### Data Integration

For the analysis and interpretation of the 2 datasets, a joint display approach will be used to visually integrate and present the mixed methods findings [39]. After all data have been collected, the quantitative results will be mapped onto the qualitative themes to examine both the similarities and divergences between patients’ reported experiences and their expressed preferences in hypothetical treatment scenarios. This joint display of qualitative and quantitative findings will facilitate a holistic interpretation, highlighting which factors most strongly influence treatment decisions and how these preferences vary across patient subgroups. The final presentation will include integrated tables and figures illustrating the convergence and complementarity of the quantitative and qualitative data.

### Ethical Considerations

This study was reviewed and determined to be exempt from full ethical review by the ethics committee of Guangdong Provincial Hospital of Traditional Chinese Medicine on November 6, 2023 (ZM2023-405). Informed consent will be obtained from all participants.

## Results

On the basis of the search strategy we formulated (Multimedia Appendix 1), a total of 18,188 articles published after 2016 were initially identified in the English- and Chinese-language databases. The outline of the semistructured interview guide for this study has been developed (Table 1). Further clinical evidence updates, qualitative interviews, and DCEs are still ongoing and will be completed by April 2026.

## Discussion

### Expected Findings

In this study, we seek to understand the treatment preferences and needs of patients with COPD regarding TCM. Our findings are anticipated to provide new insights into how patients’ personal values and experiences shape treatment choices, helping physicians tailor clinical decisions to individual characteristics and needs.

EBM is defined as “the conscientious, explicit, and judicious use of the current best evidence to make medical decisions for individual patients” [40]. True EBM prioritizes individualized patient care by integrating clinical evidence with the unique contexts, experiences, and preferences of patients [41]. However, over the years, investigators have been committed to addressing patient population and subgroup-specific issues when designing and conducting clinical studies, often at the expense of individual patient differences to achieve more generalized findings and broader application prospects. Nevertheless, contemporary research, guideline development, and even clinical decision-making continue to insufficiently reflect individual patients’ values and preferences [42,43].

The personal experience of patients is extremely important and difficult to standardize, and conclusions drawn from studies on general patient populations should not outweigh those drawn by individual patients through their own experiences. Therefore, recent advances in EBM call for a more balanced approach that integrates critical evidence appraisal with patients’ values and preferences through SDM [44]. Achieving this integration requires clinicians to invest substantial time and effort in explaining, discussing, and negotiating treatment options with patients.

Previous studies on the treatment preferences of patients with COPD have mainly focused on conventional therapies such as inhalers, pharmacotherapy, and lung transplantation [45,46]. A pilot study by our team suggested that preferences for TCM may be influenced by perceived efficacy, side effects, and cost, but these findings require validation in larger samples [29]. Moreover, previous research has rarely examined patient perceptions of specific TCM interventions or accounted for individual values, experiences, and cultural factors.

This study has several strengths. First, it comprehensively captures the preferences and needs of patients with COPD regarding TCM through an exploratory sequential mixed methods study design that allows for a comparative analysis examining the relationship between patient preferences and individual backgrounds, helping identify patterns of homogeneity or divergence in treatment inclinations. Second, the use of narrative medicine in the interviews enables a deeper exploration of patients’ true experiences and perceptions, mitigating biases that arise when patients favor previously used treatments even when there is new evidence suggesting alternatives [47]. Finally, including patients from diverse hospital backgrounds and with diverse treatment experiences ensures that multiple perspectives are represented.

However, several limitations should be acknowledged. Patient preferences may still be influenced by cognitive perceptions, social determinants, environmental factors, or previous experiences, which could limit generalizability. Future multicenter studies with larger samples are needed to validate and extend these findings.

### Dissemination

The findings will be shared at national and international conferences focused on respiratory and integrative medicine. For scientific publication, results will be submitted to peer-reviewed journals related to respiratory care, patient-centered medicine, or integrative medicine. To reach patients and caregivers, summaries will be disseminated via patient organizations, hospital newsletters, and social media channels. These efforts aim to ensure that the study results are accessible and understandable to both the scientific community and the wider public.

### Conclusions

In conclusion, this exploratory sequential mixed methods study will generate empirical evidence on the treatment preferences of patients with COPD regarding TCM, offering a foundation for SDM and patient-centered COPD care. The anticipated findings will contribute to personalized treatment strategies; inform integrative guideline development; support SDM; and, ultimately, improve patient adherence and satisfaction. Future work will extend recruitment across multiple regions and countries to enhance generalizability and explore digital health applications that integrate patient preference modeling into clinical decision support systems.

## Data Availability

Data sharing is not applicable to this article as no datasets were generated or analyzed during this study. However, collective data from future studies will be made available as supplementary files alongside the manuscript.

## Appendix

Multimedia Appendix 1 Detailed search strategy.

## Abbreviations

TCM: traditional Chinese medicine
COPD: chronic obstructive pulmonary disease
DCE: discrete choice experiment
EBM: evidence-based medicine
PRISMA: Preferred Reporting Items for Systematic Reviews and Meta-Analyses
SDM: shared decision-making
