# Supplementary material for: Patients’ Preferences Regarding Traditional Chinese Medicine for the Treatment of Chronic Obstructive Pulmonary Disease: Protocol for a Mixed Methods Study
Source: JMIR Res Protoc. 2025 Dec 2;14:e75426. doi: 10.2196/75426 (PMC12709160; doi:10.2196/75426)
Supplement: Multimedia Appendix 1 [file resprot_v14i1e75426_app1.docx]

**Detailed Search strategy**

Table S1. English search on PubMed for example.

| Type | English Search term |
| --- | --- |
| Disease | 1. "pulmonary disease, chronic obstructive"[MeSH Terms] OR "bronchitis, chronic"[MeSH Terms] OR "pulmonary emphysema"[MeSH Terms] OR chronic obstructive pulmonary disease[MeSH Terms] OR chronic obstructive lung disease[MeSH Terms] OR chronic obstructive airway disease[MeSH Terms] OR Emphysema[Title/Abstract] OR COPD[Title/Abstract] OR Chronic Obstructive Pulmonary[Title/Abstract] OR COAD[Title/Abstract] OR AECB[Title/Abstract] OR COBD[Title/Abstract] OR Chronic Obstructive Airway[Title/Abstract] OR Chronic Obstructive Lung[Title/Abstract] OR Chronic obstructive bronchopulmonary[Title/Abstract] OR Chronic obstructive respiratory[Title/Abstract] OR Chronic Airflow Obstruction[Title/Abstract] OR Chronic Airflow Obstructive[Title/Abstract] OR Chronic bronchitis[Title/Abstract] OR Pulmonary emphysema[Title/Abstract] OR Lung emphysema[Title/Abstract] OR Chronic Airflow limitation[Title/Abstract] |
| Intervention | 1. traditional chinese medicine[MeSH Terms] OR acupuncture[MeSH Terms] OR drugs, chinese herbal[MeSH Terms] OR moxibustion[MeSH Terms] OR meridians[MeSH Terms] OR auriculotherapy[MeSH Terms] OR chinese drugs, plant[MeSH Terms] OR tai chi[MeSH Terms] OR qi gong[MeSH Terms] OR Traditional Chinese Medicine[Title/Abstract] OR Chinese Drugs, Plant[Title/Abstract] OR TCM[Title/Abstract] OR Single Prescription[Title/Abstract] OR Herbs[Title/Abstract] OR Chinese Medicine Herb[Title/Abstract] OR Herbal Medicine[Title/Abstract] OR Chinese Medicine[Title/Abstract] OR Acupuncture[Title/Abstract] OR Meridians[Title/Abstract] OR Electroacupuncture[Title/Abstract] OR Moxibustion[Title/Abstract] OR Auriculotherapy[Title/Abstract] OR plum blossom[Title/Abstract] OR acupressure[Title/Abstract] OR moxa[Title/Abstract] OR point injection[Title/Abstract] OR catgut embedding[Title/Abstract] OR Tai chi[Title/Abstract] OR Qi gong[Title/Abstract] OR Tuina[Title/Abstract] OR Chinese massage[Title/Abstract] OR cupping[Title/Abstract] OR guasha[Title/Abstract] OR blood letting[Title/Abstract] |
| Study type | 1. randomized controlled trial[Publication Type] OR randomized[Title/Abstract] OR placebo[Title/Abstract] |
| **Search strategy** | 1. **AND ② AND ③** |

Table S2. Chinese search on China National Knowledge Infrastructure (CNKI) for example.

| Type | Chinese Search term |
| --- | --- |
| Disease | 1. SU%慢性阻塞性肺疾病 OR SU%慢性阻塞性肺病 OR SU%COPD OR SU%慢阻肺 OR SU%阻塞性肺病 OR SU%阻塞性肺疾病 |
| Intervention | 1. SU%中医 OR SU%中西医 OR SU%传统医学 OR SU%中药 OR SU%针刺 OR SU%灸 OR SU%针法 OR SU%刺法 OR SU%穴位 OR SU%外治 OR SU%推拿 OR SU%拔罐 OR SU%气功 OR SU%太极 |
| Study type | 1. FT%随机 |
| **Search strategy** | 1. **AND ② AND ③** |
